# Estimation of Antioxidant Levels in Saliva and Serum of Chronic Periodontitis Patients with and without Ischemic Heart Disease

**DOI:** 10.1155/2017/1965697

**Published:** 2017-07-11

**Authors:** Anahita Punj, Santhosh Shenoy, N. Suchetha Kumari, Priyanka Pampani

**Affiliations:** ^1^Department of Periodontics, A.B. Shetty Memorial Institute of Dental Sciences, Nitte University, Deralakatte, Mangalore, Karnataka 575018, India; ^2^Department of Biochemistry, K.S. Hegde Medical Academy, Nitte University, Deralakatte, Mangalore, Karnataka 575018, India

## Abstract

**Objective:**

To investigate whether there is a relationship between periodontitis and ischemic heart disease by estimation of total antioxidant status in saliva and serum.

**Materials and Methods:**

A total of 80 samples were collected and divided equally into 4 groups of healthy controls, chronic periodontitis patients, ischemic heart disease patients with periodontitis, and ischemic heart disease patients without periodontitis. Saliva and venous blood samples were collected and analyzed for levels of total antioxidant capacity, superoxide dismutase, glutathione peroxidase, and catalase.

**Results:**

There were significant (*p* < 0.05) differences in the mean serum levels of total antioxidant capacity (*p* < 0.001), superoxide dismutase (*p* < 0.001), glutathione peroxidase (*p* < 0.006), and catalase (*p* < 0.001) within the 4 groups, whereas the mean salivary levels were significant only for glutathione peroxidase (*p* = 0.001). Both of these serum and salivary antioxidant levels were lower in disease groups of IHD + CP, IHD + H, and CP as compared to healthy controls, with different patterns.

**Conclusion:**

Antioxidant capacity is significantly hampered in chronic periodontitis and ischemic heart disease patients with or without periodontitis as compared to healthy controls. The salivary and serum antioxidants may not follow the same increase or decrease as a result of increased oxidant stress due to disease.

## 1. Introduction

The periodontium is a specialized connective tissue that includes the gingiva, periodontal ligament, cementum, and alveolar bone. All these collectively act as a single unit that supports the tooth in its socket [1].

Chronic periodontitis is an infectious disease set off by Gram-negative bacteria residing in the subgingival biofilm, leading to the destruction of soft and hard tissues surrounding the teeth, and is responsible for causing tooth loss [1].

Periodontal pathogens and their products activate the host defense mechanisms and induce the formation of reactive oxygen species (ROS) or free radicals. Free radicals like O_2_^−^, H_2_O_2_, HOCL, and OH^−^ have unpaired electrons making them highly reactive. These are released by the activated PMNs and cause oxidative killing of bacteria in biofilms [2, 3]. This creates an immune response to the microorganism, which results in further release of cytokines and proinflammatory mediators (IL-1*α*, IL-1*β*, IL-6, IL-8, and TNF-*α*) causing amplification of the inflammatory process. Prolonged exposure to ROS leads to a wide spectrum of pathologic reactions in host tissue (lysis of cell membrane, DNA fragmentation, inactivation of enzymes, and activation of proteolytic enzymes resulting in degradation of specific extracellular matrix components of hyaluronic acid and proteoglycan) [4–6].

However, the release of these species outside of phagocytes is detrimental for the surrounding tissues and contributes to the pathogenesis of various inflammatory diseases, through harmful oxidative reactions [2, 3]. Cells and tissues under physiological conditions also generate ROS, which not only have an important role in the process of cell signaling and metabolic processes (Bogdan et al., 2000) [7] but also are believed to play a role in the pathogenesis of various inflammatory disorders (Davies, 1995; McCord, 2000) [8, 9] which range from periodontal diseases to systemic diseases such as diabetes mellitus and cardiovascular diseases (ischemic heart disease) [7–10].

The human body has a range of antioxidant defense mechanisms (nonenzymatic and enzymatic antioxidants) to eliminate reactive oxygen species and prevent their harmful consequences on the host [2, 3]. Antioxidant enzymes protect tissues against oxidative injury by scavenging free oxygen radicals generated by various metabolic processes, modulating the extent of inflammatory response. Antioxidant molecules are present in all body fluids and tissues. The most important intracellular enzymes which protect cells and tissues from the oxygen-derived free radicals are superoxide dismutase (SOD), which rapidly causes dismutation of the superoxide anion to hydrogen peroxide, glutathione peroxidase (GPx), which reduces hydrogen peroxide (a precursor of more potent radical species) and/or lipid hydrogen peroxides by the oxidation of reduced glutathione or S-nitrosoglutathione, and catalase (CAT), which scavenges hydrogen peroxide by converting it to oxygen and water [3].

A deficiency or decrease in antioxidant capacity increases susceptibility to oxidative stress, and the resulting damage is thought to be involved in the pathogenesis of various diseases. Previous studies have reported that periodontal disease was associated with a reduced salivary antioxidant capacity and increased oxidative damage within the oral cavity. There is increasing evidence that oxidative stress is an important contributing factor in the pathogenesis of cardiovascular disease and periodontal disease [3].

Existing evidence suggests that untreated periodontal disease increases the risk of systemic disease by inducing a prolonged systemic inflammatory response. Current literature evidence has demonstrated a modest but statistically significant association between periodontal disease and cardiovascular disease. In atherogenesis, events such as endothelial cell expression of adhesion molecules and development of fatty streaks have been postulated to be regulated, in addition to other factors, by chronic systemic inflammation [11]. Oxidative stress can result in endothelial dysfunction which is observed in atherosclerotic heart disease [12, 13].

Recently, the advances in our understanding regarding the etiology, risk factors, and progression of periodontal diseases have led to research for specific risk factors such as the antioxidant capacity [14].

Thus, the aim of this study was to investigate the total antioxidant capacity and levels of superoxide dismutase, glutathione peroxidase, and catalase in serum and saliva in chronic periodontitis patients with and without ischemic heart disease and to correlate the salivary and serum levels.

## 2. Materials and Methods

The research protocol was submitted and approved by the Ethical Committee of Nitte University, dated 06-03-2015 (Cert. number: ABSM/EC/22/2015) and was conducted in accordance with the ethical regulations of the Declaration of Helsinki.

### 2.1. Study Population

Patients from the Outpatient Department of Periodontics and Department of Cardiology were screened based on selection criteria and a sample of 80 patients were selected. Prior to the study, written informed consent was acquired from all the participants.

Patients between the ages of 25–65 years and with minimum complement of 20 teeth were included in the study. The detailed case history and medical history were recorded for all selected patients.

All the patients were screened to assess their periodontal status. The periodontal examination consisted of Loe and Silness (1963) [15] Gingival Index (GI), Probing Pocket Depth (PPD), and Clinical Attachment Level (CAL). PPD, defined as the distance between the gingival margin and the base of the pocket, was measured on six sites of the tooth (mesial, median, and distal points on the buccal and palatal aspect). Based on the level of gingival margin from the cementoenamel junction (CEJ), Clinical Attachment Level (CAL) is calculated, which is the distance from the CEJ to the base of the gingival sulcus or pocket which is measured using a Michigan “O” probe with William's markings.

Then, patients were categorized into the following four groups with 20 patients each (H: healthy group; CP: chronic periodontitis group; IHD: ischemic heart disease group without periodontitis; IHD + CP: ischemic heart disease group with periodontitis) based on the inclusion criteria mentioned below.

Patients who had no systemic diseases and were periodontally healthy with no attachment loss and probing depth <3 mm were considered as healthy controls. Patients who were diagnosed with ischemic heart disease based on clinical presentation and ECG changes by a cardiologist were included. The duration from the date of diagnosis till sample collection ranged from 6 months to 2 years for ischemic heart disease patients. Patients showing the presence of more than 30% of sites with clinical attachment loss ≥3 mm and probing depth ≥4 mm [16] measured with Michigan “O” probe with William's markings were diagnosed as having a moderate to severe form of chronic periodontitis.

Patients with a history of any antibiotic/anti-inflammatory therapy for 6 months prior to study, any known systemic disease or conditions apart from ischemic heart disease, history of smoking, tobacco consumption, and vitamin/minerals or antioxidants supplements intake during the last 3 months, and periodontal therapy 6 months prior to study and pregnant/lactating women were excluded from the study.

#### 2.1.1. Sample Collection

Participants were instructed not to eat, drink, chew gum, or brush teeth for at least 30 min before sampling. Ischemic heart disease patients were instructed to refrain from taking any medication 24 hours before sampling based on the physician's consent. Unstimulated saliva was collected from patients between 9 and 11 a.m. The patients were asked to sit comfortably and were instructed to allow the saliva to pool in the bottom of their mouth and drain it into plastic containers. After 20 minutes of rest, about 5 mL of venous blood was drawn by venepuncture from the median cubital vein and collected in plain and ethylenediaminetetraacetic acid (EDTA) blood collection plastic tubes. The collected samples were sent for biochemical analysis for estimation of total antioxidants level, superoxide dismutase, catalase, and glutathione peroxidase.

#### 2.1.2. Biochemical Analysis

The levels of superoxide dismutase (SOD), glutathione peroxidase (GPx), catalase (CAT), and total antioxidant capacity (TAC) were estimated by NBT reduction method, Rotruck method, Luck method, and phosphomolybdenum method using a spectrophotometer (Systronics Spectrophotometer 106) at wavelengths of 560 nm, 412 nm, 240 nm, and 695 nm, respectively.

### 2.2. Statistical Analysis

The data collected was compiled in an Excel sheet and analyzed using SPSS version 22 software. Mean and standard deviation (descriptive statistics) were calculated and presented in tables. Level of significance for this study was 5% with a *p* value of <0.05. Since the data did not follow normal distribution, Kruskal–Wallis test was used to compare the antioxidant levels between the four groups (healthy patients, patients with chronic periodontitis, and IHD patients with and without periodontitis). To correlate antioxidant levels in saliva and serum, Spearman's correlation coefficient was used. Bar diagrams and scatter plots were used to represent the results of the statistical analysis.

## 3. Results

Out of the 80 patients included in the study, 25 were females, while 55 were male patients. The mean age of the patients included in the study was 47.3 years. There were no menopausal women in the study.

The mean antioxidant levels in serum and saliva of healthy subjects, chronic periodontitis patients, ischemic heart disease patients, and ischemic heart disease patients with chronic periodontitis are given in all figures and tables.

**Figure 1:**
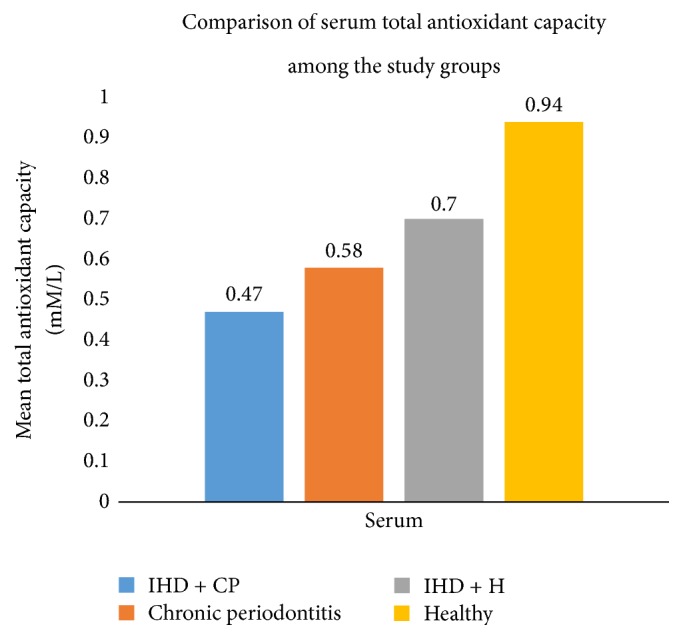


**Figure 2:**
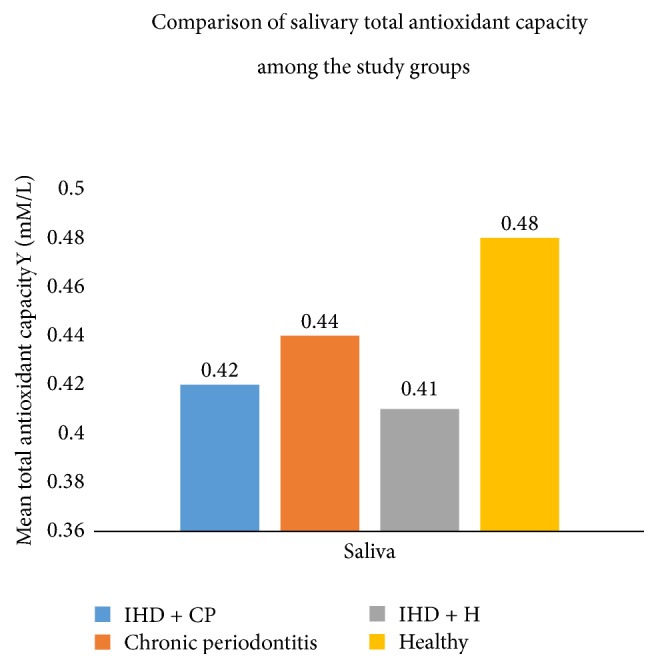


The results revealed that there were differences in the mean levels of total antioxidant capacity (TAC), superoxide dismutase (SOD), glutathione peroxidase (GPx), and catalase (CAT) within the 4 groups in both serum and saliva (Figures 1 and 2, Tables 1–4).

Results of the serum estimation (Table 1) indicated that the levels of antioxidants (TAC, CAT, and GPx) were comparatively higher in the healthy group as compared to the other 3 groups with the least values observed in the ischemic heart disease group with and without periodontitis. Serum SOD levels depicted higher levels in ischemic heart disease groups and the lowest levels in chronic periodontitis groups. All the values were significant with *p* < 0.05. Pairwise comparison of the serum levels of total antioxidant capacity (TAC), superoxide dismutase (SOD), glutathione peroxidase (GPx), and catalase (CAT) among the four groups (Table 2) further confirmed the differences present among the four groups for each of the antioxidants studied.

The mean salivary levels of the antioxidants (Table 3) showed that the antioxidants values of glutathione peroxidase were higher in the disease groups IHD + CP, CP, and IHD + H as compared to the healthy controls. The difference observed was significant with *p* < 0.05, while the values of total antioxidant capacity, superoxide dismutase, and catalase were nonsignificant. Pairwise comparison of the salivary glutathione peroxidase (GPx) levels among the four groups (Table 4) showed significant differences between CP and IHD + H, IHD + H and H, IHD + H and IHD + CP, and H and IHD + CP for GPx.

**Figure 3:**
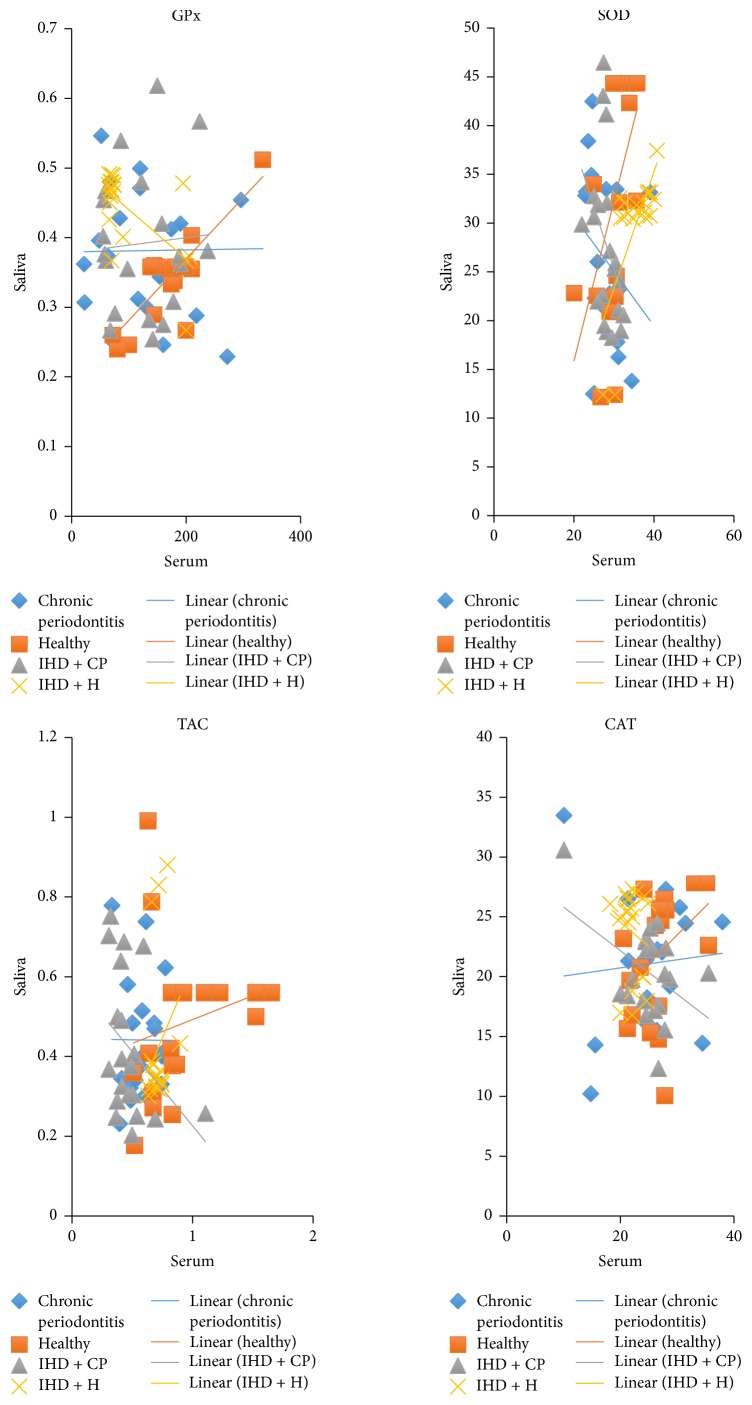


*Correlation between Serum and Salivary Antioxidants (Table 5, Figure 3).* In the ischemic heart disease group without periodontitis, a positive correlation was seen between serum and salivary SOD levels. In the healthy group, a positive correlation was seen between serum and salivary SOD, serum and salivary GPx, and serum and salivary catalase levels. On the other hand, a negative correlation was observed between serum and salivary SOD levels in the ischemic heart disease group with chronic periodontitis. The serum and salivary antioxidants in chronic periodontitis showed no correlation.

## 4. Discussion

Antioxidants like free radical scavenging enzymes (SOD) and H_2_O_2_ degrading enzyme (catalase) are of importance in providing protection to normal cells and matrix components from oxidation and have a protective role to play in our body against oxidant stress. Previous studies [2–4, 10, 17] suggested that an imbalance in antioxidant-oxidant stress makes patients more susceptible to periodontal disease as the oxygen-derived species like hydrogen peroxide (H_2_O_2_) and hydroxyl radical (OH^−^) cause cell injury, are involved in degenerative changes, and are often associated with increased peroxidative processes and linked to low antioxidant concentrations. This imbalance in the antioxidant-oxidant stress is also implicated in the pathophysiology of atherosclerotic disease process resulting in ischemic heart disease [18].

The present study showed statistically significant differences in the levels of the estimated antioxidants within the 4 groups. Comparatively higher levels of antioxidants were observed in the healthy group, which indicated that the oxidant related stress in the body is effectively balanced by the antioxidants. Lower levels of antioxidants in diseased states of chronic periodontitis and ischemic heart disease point towards the conclusion that the disease process is a result of a balance tipped in favor of the oxidants, with failure of antioxidants to balance the oxidant levels. The higher levels of SOD in IHD + H as compared to healthy controls (Table 1) probably reflect an effort of SOD to counteract the excessive oxidant stress present in IHD + H. Similarly, the higher salivary GPx levels in disease groups of IHD + CP, IHD + H, and CP as compared to the healthy controls (Table 3) could probably be a result of a compensatory increase of GPx to the oxidant stress in diseased states. The lower salivary GPx level could be due to either the increased consumption of GPx or the decreased oxidant levels in healthy saliva.

There were significant differences in the salivary TAC levels between healthy and chronic periodontitis patients (Figure 2). These findings are in accord with a study done by Diab-Ladki et al. (2003), who evaluated total antioxidant activity of saliva in periodontitis patients, which revealed that there was a significant decrease in total antioxidant activity of saliva in patients with periodontitis when compared to healthy individuals [3].

A study done by Sculley and Langley-Evans (2003) also reported similar findings and concluded that the decrease in salivary TAC could be explained by the destruction caused by the imbalance between antioxidants and ROS [2].

The results of this study for serum TAC (Figure 1) are in broad agreement with observations of Chapple et al. and Brock et al. wherein the TAC levels are decreased in the periodontitis group as compared to healthy controls [17, 19].

Kusano and Ferrari (2008) conducted a study which revealed that there was an inverse relation association between total antioxidant capacity and the number of damaged vessels and increased risk of coronary artery disease [20].

Significant differences in serum catalase were also observed between the healthy and ischemic heart disease groups which are supported by the study done by Noichri et al. (2013) [21].

Studies done by Ogunro et al. (2009) and Kumar et al. (2012) in ischemic heart disease patients revealed a significant decrease in total antioxidant levels and serum antioxidant levels (SOD and CAT), respectively, in comparison with control groups [22, 23].

Intracellular SOD (the most prominent antioxidant in mammalian tissues) is predominantly localized in the periodontal ligament in association with collagen fibrils and fibroblasts. A study by Ellis et al. [24] reported a decrease in SOD levels with the increase in probing depth; the decrease in SOD levels caused an increase in leukocytes and an increase in ROS. Depressed levels of serum antioxidants in chronic periodontitis in comparison to the healthy group can be explained by the hyperactivity of neutrophils (in chronic periodontitis) producing ROS in response to Fc *γ* receptor stimulation [7].

Catalase guards the cells from hydrogen peroxide generated within them. It plays a role in tolerance acquisition to oxidative stress in the adaptive response of cells. Lower catalase levels are seen in periodontitis [7, 10, 25].

Glutathione peroxidase is a selenium containing peroxidase and shares substrate (H_2_O_2_) with catalase. It alone can react effectively with lipid and other organic hydroperoxidases. Glutathione peroxidase provides protection against low levels of oxidant stress, whereas catalase becomes more significant in defending against severe oxidant stress [7, 10, 26, 27].

Panjamurthy et al. [28] reported that the enzymatic antioxidant activity (SOD, CAT, and GPx) in plasma, erythrocytes, erythrocyte membrane, and gingival tissue of periodontitis patients was significantly higher in the chronic periodontitis group relative to parameters found in healthy patients.

Positive correlation between serum and salivary SOD levels in the ischemic heart disease group and healthy group is in accordance with the study by Al-Rawi et al. (2008) where the salivary SOD followed serum SOD [29].

The results of this study are in contrast to studies done by Moore et al. [30] and Chapple et al. [17], which showed no significant differences in the levels of antioxidants in periodontitis patients in comparison with control groups.

Tsai et al. [31] found that there was no difference in the salivary GPx activity in periodontitis patients relative to controls. This is in contrast to the present study, where the salivary GPx levels are significantly higher in disease groups to compensate the oxidant stress. Kim et al. [4] in their research found that the salivary total antioxidant level was higher in severe chronic periodontitis patients than in the healthy or gingivitis controls before scaling and root planning, whereas SOD activity of the periodontitis patients was lower than of the controls at each time [20].

Wei et al. measured the levels of superoxide dismutase in serum, saliva, and GCF in chronic periodontitis patients before and after periodontal therapy and found that the levels of superoxide dismutase (SOD) were significantly higher in the chronic periodontitis group than in the healthy control group. After periodontal therapy, SOD levels significantly decreased compared to basal levels (*p* < 0.05) in serum, saliva, and GCF [32].

This study is the first to compare the serum and salivary levels of the antioxidants (TAC, SOD, catalase, and glutathione levels) within the groups of healthy subjects, chronic periodontitis patients, and ischemic heart disease patients with or without periodontitis.

## 5. Limitations

This study had a small sample size. A larger sample size and better selection criteria with the use of adjusted models for confounding factors could have been used. All these limitations warrant further studies of prospective, interventional, and experimental study designs.

## 6. Conclusion

The present study showed a statistically significant reduction in total antioxidant capacity, superoxide dismutase, catalase, and glutathione peroxidase in patients with chronic periodontitis and ischemic heart disease with or without periodontitis when compared to healthy patients.

Overall, there was a significant correlation between the salivary and serum levels of SOD in the four antioxidant parameters between the four groups, although few parameters showed negative or no correlation. This indicates that probably saliva may not serve as a substitute to serum as a diagnostic sample for antioxidant estimation, as there is no uniformity in the correlation of serum and salivary antioxidant levels observed in this study.

Thus, the antioxidant capacity is significantly hampered in chronic periodontitis and ischemic heart disease patients with or without periodontitis.

However, the oxidant stress in chronic periodontitis generated by free radicals may result in endothelial dysfunction of the blood vasculature, predisposing to atherosclerotic plaque formation and increasing predisposition to ischemic heart disease. It is difficult to conclude to what extent chronic periodontitis has an effect on ischemic heart disease, as depicted by the levels of antioxidants in the human body. However, it is important to note that the reduced antioxidant capacity may result either from an increase in oxidative stress or from lower antioxidant defence status.

Long-term studies are required to justify whether antioxidant levels can be used as a prognostic or diagnostic marker to predict the association between chronic periodontitis and ischemic heart disease.

## Figures and Tables

**Table 1:** Comparison of serum levels.

Antioxidant	Group	*N*	Mean (SD)	Median (Q1–Q3)	*H*-value^#^ (df)	*p* value
Serum TAC (mM/L)	IHD + CP	20	0.47 (0.17)	0.42 (0.37–0.51)	37.33 (3)	<0.001^*∗*^
	CP	20	0.58 (0.13)	0.57 (0.48–0.72)		
	IHD + H	20	0.70 (0.05)	0.70 (0.66–0.73)		
	H	20	0.94 (0.36)	0.83 (0.66–1.19)		

Serum SOD (U/mg Hb)	IHD + CP	20	28.17 (2.62)	28.15 (26.28–30.35)	33.27 (3)	<0.001^*∗*^
	CP	20	27.72 (4.20)	26.63 (24.65–30.79)		
	IHD + H	20	35.70 (3.72)	36.59 (32.62–38.95)		
	H	20	30.34 (3.93)	30.51 (28.71–33.84)		

Serum GPx (U/mg Hb)	IHD + CP	20	125.10 (59.63)	129.00 (62.00–173.50)	12.280 (3)	0.006^*∗*^
	CP	20	156.20 (84.40)	143.00 (75.00–219.50)		
	IHD + H	20	96.50 (53.37)	70.00 (68.00–86.00)		
	H	20	167.70 (56.21)	174.00 (140.00–199.25)		

Serum CAT (U/mg Hb)	IHD + CP	20	25.02 (4.76)	25.47 (24.11–27.53)	18.292 (3)	<0.001^*∗*^
	CP	20	25.58 (6.73)	26.11 (22.17–30.03)		
	IHD + H	20	22.01 (1.69)	21.9 (21.08–23.60)		
	H	20	26.71 (4.16)	26.80 (23.65–27.81)		

^#^Kruskal–Wallis test; ^*∗*^*p* < 0.05, statistically significant.

**Table 2:** Pairwise comparison using Mann–Whitney *U* test (serum).

Dependent variable	(*I*) condition	(*J*) condition	Mean difference (*I* − *J*)	95% confidence interval		*U* statistic^#^	*p* value
				Lower	Upper		
Serum TAC (mM/L)	CP	IHD + CP	0.10	−0.07	0.29	99.50	0.007^*∗*^
		IHD + H	−0.12	−0.30	0.06	103.00	0.009^*∗*^
		H	−0.35	−0.54	−0.17	61.50	<0.001^*∗*^
	IHD + H	IHD + CP	0.23	0.04	0.41	27.00	<0.001^*∗*^
		H	−0.23	−0.41	−0.05	130.00	0.058 (NS)
	H	IHD + CP	0.46	0.28	0.65	27.00	<0.001^*∗*^

Serum SOD (U/mg Hb)	CP	IHD + CP	−0.45	−3.59	2.69	163.00	0.317 (NS)
		IHD + H	−7.97	−11.12	−4.83	36.00	<0.001^*∗*^
		H	−2.61	−5.76	0.52	118.0	0.027^*∗*^
	IHD + H	IHD + CP	7.52	4.37	10.66	23.00	<0.001^*∗*^
		H	5.36	2.21	8.50	59.50	<0.001^*∗*^
	H	IHD + CP	2.16	−0.98	5.30	123.00	0.037^*∗*^

Serum GPx (U/mg Hb)	CP	IHD + CP	31.10	−24.23	86.43	164.50	0.337 (NS)
		IHD + H	59.70	4.36	115.03	120.50	0.031^*∗*^
		H	−11.50	−66.83	43.83	171.00	0.433 (NS)
	IHD + H	IHD + CP	−28.60	−83.93	26.73	165.00	0.343 (NS)
		H	−71.20	−126.53	−15.86	68.50	<0.001^*∗*^
	H	IHD + CP	42.60	−12.73	97.93	119.50	0.029^*∗*^

Serum CAT (U/mg Hb)	CP	IHD + CP	0.56	−3.45	4.59	180.50	0.598 (NS)
		IHD + H	3.57	−0.45	7.59	91.50	0.003^*∗*^
		H	−1.12	−5.15	2.89	187.50	0.735 (NS)
	IHD + H	IHD + CP	−3.00	−7.03	1.01	71.50	0.001^*∗*^
		H	−4.70	−8.72	−0.67	59.00	<0.001^*∗*^
	H	IHD + CP	1.69	−2.33	5.71	164.00	0.330 (NS)

^#^Mann–Whitney *U* test; ^*∗*^*p* < 0.05, statistically significant; *p* > 0.05, nonsignificant (NS).

**Table 3:** Comparison of salivary levels.

Antioxidant	Group	*N*	Mean (SD)	Median (Q1–Q3)	*H*-value^#^ (df)	*p* value
Saliva TAC (mM/L)	IHD + CP	20	0.42 (0.17)	0.37 (0.26–0.60)	3.921 (3)	0.270 (NS)
	CP	20	0.44 (0.14)	0.40 (0.33–0.50)		
	IHD + H	20	0.41 (0.18)	0.34 (0.32–0.38)		
	H	20	0.48 (0.18)	0.53 (0.36–0.56)		

Saliva SOD (U/mg protein)	IHD + CP	20	27.60 (8.37)	25.60 (20.70–32.03)	4.939 (3)	0.176 (NS)
	CP	20	26.34 (9.08)	24.61 (18.72–33.47)		
	IHD + H	20	30.10 (6.25)	31.51 (30.58–32.89)		
	H	20	32.78 (11.76)	33.15 (22.48–44.33)		

Saliva GPx (*μ*g/g protein)	IHD + CP	20	0.39 (0.10)	0.37 (0.29–0.46)	17.230 (3)	0.001^*∗*^
	CP	20	0.38 (0.09)	0.38 (0.30–0.46)		
	IHD + H	20	0.44 (0.05)	0.46 (0.40–0.47)		
	H	20	0.34 (0.06)	0.35 (0.30–0.35)		

Saliva CAT (U/mg protein)	IHD + CP	20	20.34 (3.92)	20.02 (17.72–22.82)	7.449 (3)	0.059 (NS)
	CP	20	21.11 (5.71)	21.49 (15.71–25.50)		
	IHD + H	20	23.74 (3.71)	25.48 (20.00–26.51)		
	H	20	21.91 (5.24)	23.75 (16.98–26.24)		

^#^Kruskal–Wallis test; ^*∗*^*p* < 0.05, statistically significant; *p* > 0.05, nonsignificant (NS).

**Table 4:** Pairwise comparison using Mann–Whitney *U* test.

Dependent variable	(*I*) condition	(*J*) condition	Mean difference (*I* − *J*)	Std. error	95% confidence interval		*U* statistic^#^	*p* value
					Lower	Upper		
Saliva GPx (*μ*g/g protein)	CP	IHD + CP	−0.01	0.02	−0.08	0.05	194.50	0.882 (NS)
		IHD + H	−0.06	0.02	−0.13	0.01	123.00	0.037^*∗*^
		H	0.04	0.02	−0.02	0.11	144.0	0.128 (NS)
	IHD + H	IHD + CP	0.05	0.02	−0.01	0.12	121.5	0.03^*∗*^
		H	0.10	0.02	0.03	0.17	39.50	<0.001^*∗*^
	H	IHD + CP	−0.05	0.02	−0.12	0.01	124.50	0.04^*∗*^

^#^Mann–Whitney *U* test; ^*∗*^*p* < 0.05, statistically significant; *p* > 0.05, nonsignificant (NS).

**Table 5:** Correlation between serum and salivary levels of TAC, SOD, GPx, and CAT.

Parameters	Condition			
	IHD + CP	CP	IHD + H	H
TAC				
Correlation coefficient	−0.399	0.203	0.261	0.374
*p* value	0.081 (NS)	0.390 (NS)	0.266 (NS)	0.104 (NS)
SOD				
Correlation coefficient	−0.487	−0.403	0.680	0.568
*p* value	0.030^*∗*^	0.078 (NS)	0.001^*∗*^	0.009^*∗*^
GPx				
Correlation coefficient	−0.042	−0.196	−0.341	0.467
*p* value	0.860 (NS)	0.409 (NS)	0.141 (NS)	0.038^*∗*^
CAT				
Correlation coefficient	−0.114	0.232	−0.026	0.475
*p* value	0.631 (NS)	0.326 (NS)	0.912 (NS)	0.034^*∗*^

Spearman's correlation test; ^*∗*^*p* < 0.05, statistically significant; *p* > 0.05, nonsignificant (NS).
